# Cough sound spectro-temporal analysis and automated detection using Vision Transformers

**DOI:** 10.1177/20552076251394623

**Published:** 2025-12-02

**Authors:** Keming Tan, Jacky Smith, Patrick Gaydecki

**Affiliations:** 1 Department of Electrical and Electronic Engineering, 5292 The University of Manchester, Manchester, UK; 2 Division of Immunology, 5292 Immunity to Infection and Respiratory Medicine, The University of Manchester, Manchester, UK; 3 5293 Manchester University NHS Foundation Trust, Manchester, UK

**Keywords:** Cough detection, vision transformer, deep learning, spectrogram, respiratory disease, automated monitoring, machine learning, clinical validation

## Abstract

**Objective:**

Cough is a key symptom in respiratory diseases, yet its clinical assessment remains challenging, often relying on subjective questionnaires or inefficient manual cough counting. Existing automated cough detection algorithms have limited generalisability due to a lack of validation on large, diverse datasets. This study aimed to develop and evaluate a fully automated cough detection system using spectro-temporal analysis and a Vision Transformer (ViT) model.

**Methods:**

A total of 231 annotated 24-hour cough recordings across 9 diagnostic categories from the RaDAR database were analysed. Recordings were segmented and converted into spectrograms using different short-time Fourier transform settings. Data were split subject-wise into training, validation, and test sets to prevent data leakage. The ViT model was fine-tuned in two stages: a pilot stage using 10% of the data to determine optimal spectrogram parameters, followed by full-scale training on the remaining data.

**Results:**

Spectrogram configuration significantly affected performance, with 750 ms segment duration, 128-point frame size, and 32-point hop identified as optimal. With these parameters, the model achieved an F1 score of 85.02%, sensitivity of 83.64%, precision of 86.44%, and specificity of 99.67% on the test set. Diagnostic category-wise analysis showed high F1 scores in interstitial lung disease (90.83%), chronic obstructive pulmonary disease (89.60%), asthma (88.13%) and chronic cough (85.32%).

**Conclusions:**

Vision Transformers with optimised spectrogram preprocessing enable accurate, scalable cough detection across diverse populations, performing comparably to popular convolutional neural networks on a larger, more diverse, and more challenging dataset. These findings support the use of ViT-based systems for objective, automated cough monitoring in clinical practice.

## Introduction

Cough is one of the most common reasons individuals seek medical attention, imposing a significant burden on public health systems worldwide.^1^ It not only serves as a symptom of underlying respiratory conditions but also profoundly impacts quality of life by causing physical discomfort, social embarrassment and psychological distress.^2^ Chronic cough can disrupt daily activities, impair sleep and lead to complications such as musculoskeletal pain, urinary incontinence^3^ and even rib fractures.^4^

Traditionally, subjective methods like the visual analogue scale (VAS),^5^ Leicester Cough Questionnaire (LCQ)^6^ and cough-specific quality of life questionnaire (CQLQ)^7^ have been used to evaluate cough severity and impact on the life. However, these approaches have drawbacks, including recall bias and variability in symptom perception, which lead to inconsistent data.^8^ To address these limitations, an objective assessment of cough is essential for monitoring disease and evaluating treatment efficacy.^9,10^

Cough frequency offers a reliable metric for objective assessment, providing a direct, quantifiable measure of symptom burden that correlates moderately with perceived cough severity and treatment effectiveness.^11^ The data can be captured non-invasively using audio recordings or simple sensors, allowing for continuous monitoring without significantly impacting patients’ daily activities.^12^

However, assessing cough frequency presents challenges, particularly the need for extended recording periods, at least 24 hours, to capture diurnal variations.^13^ Traditionally, analysing such recordings involved human listeners manually counting each cough, which is both time-consuming and labour-intensive.^14^ Despite advances in signal processing techniques to reduce data volume and recording length, considerable effort is still required. It is expensive and limits objective assessments of cough severity in clinical practice, symptom measurements in clinical studies and efficacy tests of novel antitussive therapy research.^15^

Over the past decade, several cough-detection systems have been developed and validated, including Hull Automatic Cough Counter (HACC),^16^ Leicester Cough Monitor (LCM)^12,17^ and VitaloJAK,^15,18^ which have become well-established in cough-related research. HACC was among the first to combine digital signal processing for feature extraction with neural-network-based cough identification. Although its neural-network architecture was rudimentary by modern standards and its performance relatively modest, HACC pioneered the use of machine-learning techniques for cough detection. LCM utilised a hidden Markov model (HMM) classifier; despite now being considered outdated, its performance remains comparable to recent systems through careful manual calibration. VitaloJAK focused solely on digital signal processing (DSP) methods to truncate recordings for manual cough counting, achieving efficient data reduction with minimal missed events.

Recent studies have embraced machine-learning and deep-learning approaches to enhance automated cough detection. Two widely used platforms, SIVA^19^ and Hyfe,^20,21^ utilising deep neural networks within fully automated workflow to minimise human intervention, demonstrating high detection accuracy in several clinical studies. Nonetheless, these systems lack detailed disclosures of their feature-extraction techniques and classifier architectures.

Other notable work in recent years consistently highlights the preference of convolutional neural networks (CNNs) as the architecture of choice for automated cough detection, owing to their superior capacity to extract and process audio features represented as spectral images. Equally significant is the trend towards portable, minimally invasive devices, such as smartphones, smartwatches and smart hearables, that facilitate continuous, real-world health monitoring while reducing patient burden.^22–29^

A recurring limitation across these studies is the reliance on relatively small or narrowly focused datasets. Furthermore, many datasets are crowdsourced or drawn from public repositories, introducing heterogeneity in recording devices and formats that can compromise data quality and generalisability. The noticeable class imbalance, where non-cough sounds vastly outnumber cough events, also predisposes models to majority-class bias. Finally, evaluation metrics such as accuracy, sensitivity and specificity are frequently reported without accounting for dataset imbalance, potentially yielding misleading appraisals of model performance.^30–33^

A further challenge lies in the analysis of cough-sound features. Most cough detection studies partition recordings into short segments and represent them using Fourier-based transforms, such as spectrograms,^22,24,27,29^ mel-spectrograms^25,28^ and mel-frequency cepstral coefficients (MFCCs).^23^ While many researchers have adopted parameterisation schemes developed for speech signals, few have systematically optimised parameters specifically for cough audio (e.g. segment duration, short-time Fourier transform (STFT) frame length and hop size).^30,32^ These parameters critically shape the resulting spectro-temporal representations and, consequently, can substantially affect the classification accuracy of machine learning models trained on cough-feature images.

Given these limitations, there is a pressing need for a thorough and systematic evaluation of spectrogram parameterisation specifically tailored to cough audio, rather than relying on configurations developed for speech signals. Optimising these parameters is essential to maximise the performance of deep learning models in cough detection. Equally important is the development of a standardised, large-scale and diverse dataset that reflects real-world variability in recording conditions, devices and patient populations. Such a dataset would enable robust training and fair benchmarking of models, facilitating generalisable and clinically relevant applications.^31,33^ This study aims to address both gaps, by systematically analysing spectro-temporal parameter choices and introducing a well-curated dataset, to support the development of reliable, scalable and practical automated cough-detection systems.

## Materials and methods

### Data source

All cough recordings used in this study were obtained from the RaDAR database,^34^ which were acquired and processed using the VitaloJAK cough monitor system.^35^ The VitaloJAK monitor, manufactured by Vitalograph Ltd specifically for cough monitoring, comprises an ambulatory recording device worn in a belt pouch, a lapel microphone, and a contact microphone affixed to the upper chest for 24-hour continuous audio capture. The device is CE-marked and has FDA 510(k) clearance. Recordings are stored in stereo WAV format at an 8 kHz sampling rate and 16-bit depth; one channel corresponds to sounds from the lapel microphone and the other to those from the contact microphone. The VitaloJAK DSP algorithm^15^ is employed in each recording to eliminate most silent and non-cough sounds, reducing the average duration to approximately 1.5 hours per 24-hour recording period. Trained analysts listen to the recordings and inspecting the audio waveform using audio editing software to identify, annotate and count all cough events.

RaDAR database is established by the University of Manchester to collect anonymised acoustic recordings to facilitate the further development and testing of software algorithms for the analysis and reporting of cough parameters. All experimental protocols were approved by North West – Haydock Research Ethics Committee, and all methods were performed in accordance with relevant guidelines and regulations. All participants had provided written informed consent for their cough recording data to be used for the development of cough detection algorithms (REC Reference 13/NW/0451). The data used in this study were obtained from the RaDAR database with permission. Access to the datasets was granted in accordance with the terms and conditions set by RaDAR.

For this study, 231 recordings from the RaDAR database were selected, and only data from the contact microphone were used for model development. The contact microphone predominantly captures sounds originating from the wearer's torso and is less susceptible to ambient noise and other people's speech. This feature addresses privacy concerns and inherently mitigates sound quality degradation due to facial coverings. The recordings encompass nine diagnostic categories: chronic cough, chronic obstructive pulmonary disease (COPD), asthma, interstitial lung disease (ILD), lung cancer, paediatric conditions, bronchiectasis, cystic fibrosis and healthy volunteers.

The participants’ demographic data are presented in Table 1, and the cough recording statistics of each diagnostic category are shown in Table 2, including the number of subjects in a category, average cough recording length (after applying silence removal algorithm) and average cough count. Table 2 also reports cough counts per hour, calculated as the total cough count divided by the shortened recording duration in hours. This metric indicates the density of cough events within a recording and is crucial for assessing class imbalance between cough and non-cough events, since non-cough durations dominate overall recording time. It should not be confused with actual cough frequency (hourly rate), which is defined as the total cough count over a full 24-hour session.

**Table 1.** Cough recording demographic data.

Diagnosis	Male	Female	Age	Age unknown
Asthma	14	32	28.5 (18–70)	
Bronchiectasis	5	2	71.6 (68–75)	
Chronic cough	20	47	60.4 (28–81)	
Cystic fibrosis	1	4	34.0 (34–34)	4
COPD	13	11	72.7 (53–84)	
Healthy volunteer	9	6	43.4 (20–74)	1
ILD	27	12	69.8 (19–85)	
Lung cancer	9	16	65.0 (58–69)	21
Paediatrics	2	1	10.3 (5–14)	
Total	100	131	54.9 (5–85)	26

COPD: chronic obstructive pulmonary disease; ILD: interstitial lung disease.

**Table 2.** Cough recording statistics.

Diagnosis categories	Number of subjects	Cough recording length (seconds)	Cough count	Cough count per hour
Asthma	46	6887 (917–17031)	77 (8–442)	53.6
Bronchiectasis	7	5180 (1315–13693)	394 (201–546)	453.7
Chronic cough	67	6988 (1203–17482)	605 (15–3691)	371.3
Cystic fibrosis	5	8191 (3375–17129)	386 (7–860)	218.7
COPD	24	5216 (1497–9740)	336 (33–1041)	291.0
Healthy volunteer	15	7366 (2497–12272)	13 (0–59)	9.0
ILD	39	6953 (1502–16851)	406 (19–1737)	262.8
Lung cancer	25	5564 (1862–10554)	430 (37–1089)	327.4
Paediatrics	3	9147 (1533–17629)	1075 (22–2703)	443.0
Total	231	6648 (917–17629)	376 (0–3691)	253.2

COPD: chronic obstructive pulmonary disease; ILD: interstitial lung disease.

### Study design

**Figure 1.**
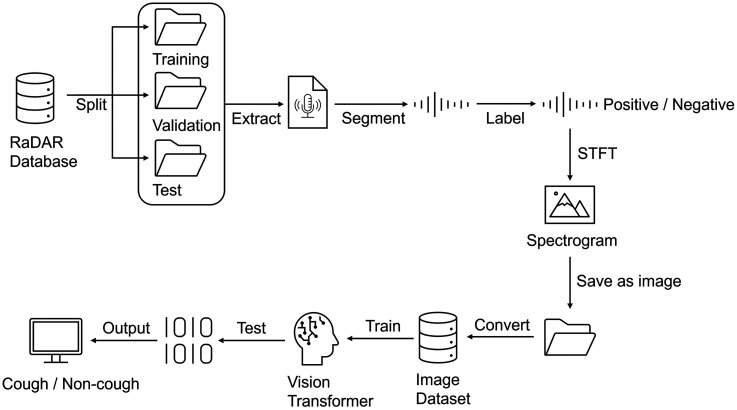
Simplified study workflow design.

The study comprised two principal stages: data processing and model development. In the data-processing stage, recordings were first split into training, validation and test sets, then segmented into short clips. Each clip was converted into a spectrogram using a variety of parameters and labelled as either a cough or non-cough sample. The model-development stage unfolded in two steps: a pilot fine-tuning, in which the model was trained, validated and tested on a 10% subset of data generated with different STFT settings to identify the optimal parameters; and a full development stage, in which the model's performance was assessed on the entire dataset using the selected STFT configuration. The workflow is illustrated in Figure 1.

#### Data split

To ensure clinically meaningful and generalisable model performance, a subject-based dataset splitting strategy was adopted. In contrast to conventional sample-based approaches, which randomly assign spectrograms extract from all recordings to subsets, this method groups all spectrogram images from a single subject into the same subset: either training (60%), validation (20%) or testing (20%). By avoiding overlap in subject identity across splits, the strategy enables the model's performance to be tested under conditions that more closely resemble deployment in real-world clinical settings. It also facilitates analysis of how well the model performs across diverse patients, diagnostic categories and demographic groups, an essential consideration in medical applications, where intersubject variability, such as differences in age, sex or underlying pathology, can significantly influence the acoustic properties of cough sounds. This subject-level split therefore supports a more robust and unbiased evaluation of model reliability and applicability in heterogeneous patient populations.

To approximate representativeness in all subsets, several rules were applied: firstly, every diagnostic category was guaranteed inclusion in all three splits, with at least one subject from small cohorts (e.g. the three-member paediatric group) assigned to training, validation and test sets alike. Secondly, within each category, subjects were allocated to the subsets in near-exact adherence to the 60/20/20 ratio, employing simple rounding rules to accommodate uneven cohort sizes. Thirdly, to preserve demographic balance, the male-female ratio observed in each diagnostic group was reproduced across all splits by proportionally sampling subjects of each sex. Finally, the average cough count per hour per category were equalised between subsets, preventing class dominance and ensuring the model encounters a uniform mixture of examples during learning. Together, these measures minimise sampling bias, reduce overfitting risk and facilitate a robust evaluation of model generalisation across diverse clinical and demographic contexts. As a result, Table 3 shows the recording splits in each subset.

**Table 3.** Cough count per hour and sex ratio in training, validation and test sets.

	Training		Validation		Test	
Diagnostic category	Count per hour	Sex ratio (M:F)	Count per hour	Sex ratio	Count per hour	Sex ratio
Asthma	53.5	8:20	53.8	3:6	53.6	3:6
Bronchiectasis	559.7	3:1	305.8	1:1	325.0	1:0
Chronic cough	371.4	12:28	370.9	4:9	371.3	4:10
Cystic fibrosis	266.7	0:3	191.0	0:1	103.0	1:0
COPD	291.1	7:7	290.4	3:2	291.2	3:2
Healthy volunteer	8.0	5:4	10.6	2:1	10.6	2:1
ILD	262.8	16:7	262.9	5:3	262.6	6:2
Lung cancer	327.2	5:10	327.5	2:3	328.0	2:3
Paediatrics	1175.0	0:1	52.0	1:0	102.0	1:0
Total	260.4	56:81	242.2	21:26	243.4	23:24

COPD: chronic obstructive pulmonary disease; ILD: interstitial lung disease.

#### Data segmentation and categorisation

Following dataset splitting, cough recordings were segmented into short clips via sliding windows prior to spectrogram generation. Because a cough may last as long as 500 ms, window lengths should be at least 500 ms to safely capture most cough events; yet overly long windows risk incorporating irrelevant sounds, background noise, speech or adjacent coughs, thereby obscuring the cough's spectral signature and increasing both data dimensionality and computational cost. To strike a balance between complete event coverage and efficiency, three window sizes were evaluated, 500, 750 and 1 000 ms, each applied with 50% overlap, corresponding to hop sizes of 250, 375 and 500 ms, respectively. The overlap ensures that most of any cough event appears intact in at least one segment, reducing boundary truncation.

For labelling, we defined a standard cough reference duration of 500 ms. A segment was designated as a ‘cough’ only if its overlap with the reference window met or exceeded a prescribed threshold:

- 500 ms segments require at least 50% overlap (≥ 250 ms) with the defined window;
- 750 ms segments require at least 75% overlap (≥ 375 ms) and
- 1000 ms segments demand full 100% overlap (500 ms), guaranteeing the entire reference window is included.

**Figure 2.**
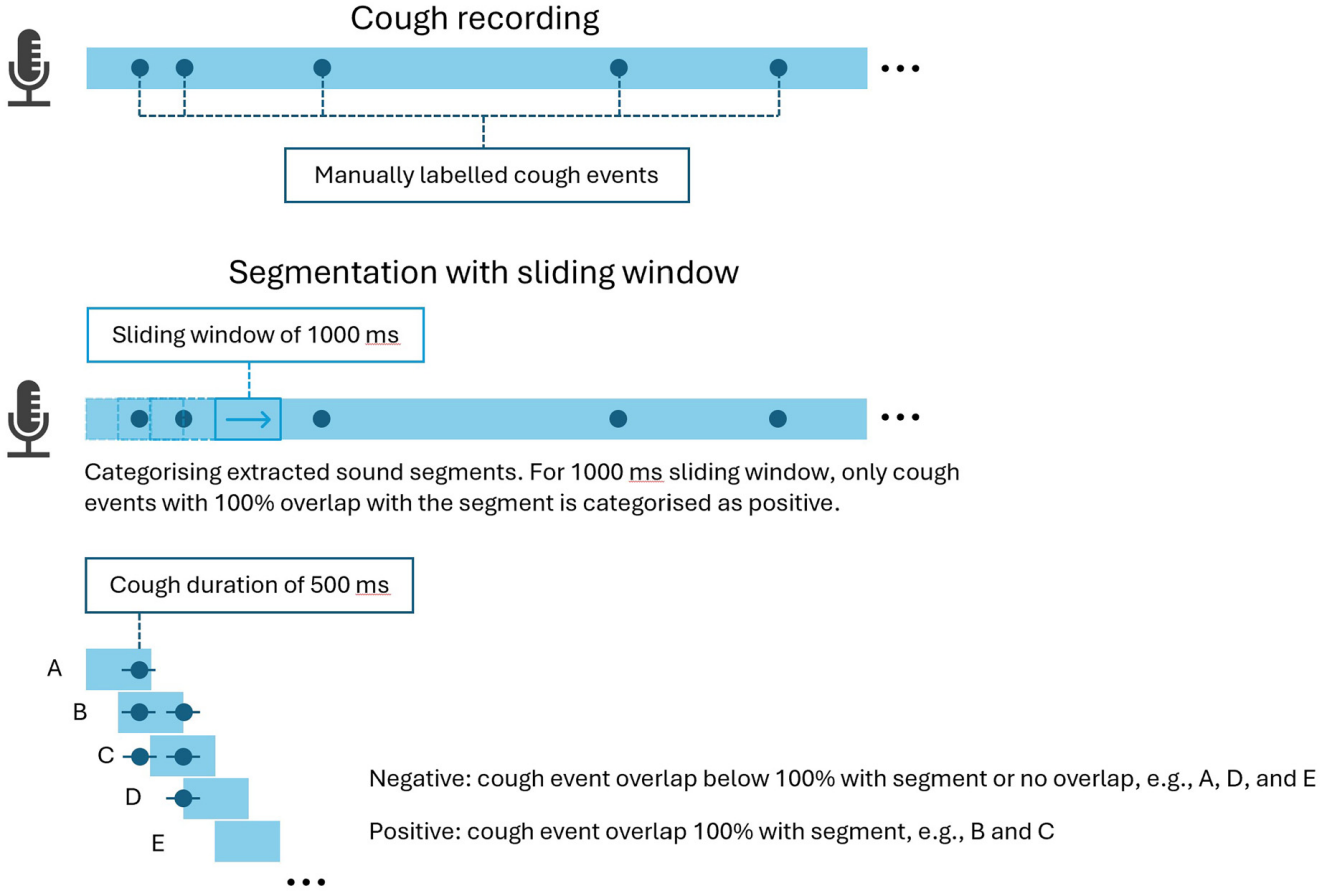
Cough segmentation and categorisation illustration (1000 ms).

Figure 2 illustrates the cough categorisation process using 1000 ms segment as an example. By employing a uniform reference duration and overlap-based labelling rule, this method mitigates the misclassification of truncated coughs and establishes a consistent fundamental for evaluating how segment length influences detection performance.

#### Spectrogram parameterisation

The selection of spectrogram parameters plays a critical role in preserving the temporal and spectral characteristics of cough events while maintaining compatibility with machine learning model input dimensions. Key parameters include sampling rate, audio duration, discrete Fourier transform (DFT) frame length and hop size, and window function, each of which affects how cough features such as explosive bursts and phonation are represented. In this study, parameter settings were carefully tailored to balance resolution, computational efficiency and model input compatibility.

All cough recordings were sampled at 8 kHz, setting the Nyquist limit at 4 kHz and defining the frequency range of interest. Frame sizes of 64, 128 and 256 samples (corresponding to 8, 16 and 32 ms) were chosen to ensure adequate temporal resolution, especially for resolving the explosive phase of coughs, which typically lasts approximately 50 ms. These frame lengths also yield frequency bin spacings between 31.25 and 125 Hz, allowing for representation of both broadband bursts and lower-frequency resonances.

Hop sizes were set to either 50% or 25% of the frame length, balancing temporal granularity and computational load. Smaller hop sizes (greater overlap) improve time resolution but increase spectrogram width and processing requirements. The number of DFT points was matched to the frame length to avoid zero-padding and utilise radix-2 FFTs for computational efficiency. Window functions Hann was chosen conventionally for its balanced trade-offs between side-lobe attenuation and main-lobe width.

It yielded 18 spectrogram configurations varied in resolution and dimensions, with heights (frequency bins) ranging from 33 to 129 pixels and widths (time frames) ranging from 30 to 497, depending on segment duration and hop size. These sizes were chosen to approximate the 224 × 224 pixels input resolution required by common image classification models, minimising the need for excessive resizing and preserving the integrity of cough-specific visual patterns. The diverse spectrogram configurations evaluated in this study provided a rich basis for identifying optimal representations for downstream classification tasks. The parameter combinations are shown in Table 4.

**Table 4.** Spectrogram parameter combinations.

Segment duration (ms)	Frame size	Hop size	Sample number	Frame resolution (ms)	Frequency bin spacing (Hz)	Image width	Image height
500	64	32	4000	8	125	124	33
500	128	64	4000	16	62.5	61	65
500	256	128	4000	32	31.25	30	129
500	64	16	4000	8	125	247	33
500	128	32	4000	16	62.5	122	65
500	256	64	4000	32	31.25	59	129
750	64	32	6000	8	125	186	33
750	128	64	6000	16	62.5	92	65
750	256	128	6000	32	31.25	45	129
750	64	16	6000	8	125	372	33
750	128	32	6000	16	62.5	184	65
750	256	64	6000	32	31.25	90	129
1000	64	32	8000	8	125	249	33
1000	128	64	8000	16	62.5	124	65
1000	256	128	8000	32	31.25	61	129
1000	64	16	8000	8	125	497	33
1000	128	32	8000	16	62.5	247	65
1000	256	64	8000	32	31.25	122	129

#### Spectrogram image conversion and dataset organisation

To prepare the 16-bit mono RaDAR audio recordings for use with standard deep learning architectures, particularly common image classification models expecting 24-bit RGB input (three 8-bit channels), a spectrogram conversion process was implemented. While this format conversion reduces the native dynamic range, it retains the spectro-temporal patterns essential for cough classification. The following steps were performed to ensure perceptual relevance, numerical stability and full compatibility with vision models.

First, the magnitude of the short-time Fourier transform (STFT) was calculated to capture the amplitude envelope. The resulting values were then transformed to a logarithmic (decibel) scale to compress the dynamic range and enhance low-amplitude features of clinical interest. Values were clipped to the range of −80 to 0 dB to suppress background noise and irrelevant low-energy components. These were subsequently normalised to the [0, 1] range and converted to 8-bit integers (0–255) to match image processing standards. The jet colormap was applied, mapping intensity values to RGB colours for improved visual distinction of cough-related patterns. Spectrogram images were resized to 224 × 224 pixels using bicubic interpolation and saved in PNG format, preserving spectral detail and ensuring compatibility with common vision transformer architectures.

This process ensures each spectrogram accurately represents the time–frequency characteristics of cough events while aligning with the input requirements of pretrained visual models.

To support reproducible training and efficient evaluation, the generated spectrograms were organised into a structured five-level folder hierarchy:

- Spectrograms/Root directory containing all image data
- [Diagnostic_Category]/Subfolders named by clinical condition (e.g. Asthma, COPD, Healthy Volunteers)
- [Subject_ID]/Anonymised participant folders for subject-wise separation
- positives/ and negatives/ Subfolders distinguishing cough and non-cough images
- [segment_index].png Filenames reflect the segment's index in the original audio, enabling back-tracing for validation or audit

This design ensures compatibility with image classification models while maintaining the integrity, traceability and clinical relevance of the original cough recordings.

#### Classification model

The study used the Vision Transformer model (ViT)^36^ instead of the widely used CNN architecture models in this study for cough spectrogram image classification. Although CNNs excel at detecting local patterns and building hierarchical features through successive layers of small, shared filters, they must gradually enlarge their receptive fields to capture long-range dependencies, an approach that can dilute global context and require deep stacks of layers to relate distant regions of an image. By contrast, the ViT reframes an image as a sequence of non-overlapping patches, each linearly projected into an embedding space and augmented with learnable positional encodings. Feeding this sequence (with a special ‘class’ token prepended) into a standard Transformer encoder allows every patch to attend directly to every other, so long-range, spectro-temporal dependencies in a cough spectrogram can be modelled in a single operation rather than through many layers of convolution.

Each Transformer block alternates multi-head self-attention, which learns multiple relationships between patches in parallel, with position-wise feed-forward layers, all wrapped in residual connections and layer normalisation. The final hidden state of the class token then summarises the entire spectrogram for classification. After large-scale pretraining to learn rich representations, ViT can be fine-tuned on smaller, cough-specific datasets with relatively few steps. This capacity to directly integrate global context from the outset makes ViT particularly well-suited for cough spectrogram detection, where relevant acoustic features often span wide time-frequency regions and must be related across the entire image.

The base model for this study was the publicly available google/vit-base-patch16-224 Vision Transformer from the Hugging Face Hub,^37^ initialised with weights pretrained on ImageNet-21k^38^ and further fine-tuned on ImageNet-1k.^39^ Each 224 × 224-pixel input spectrogram, converted to three-channel RGB, was split into non-overlapping 16 × 16 patches, linearly projected into 768-dimensional embeddings, prepended with a learnable [CLS] token, and augmented with fixed positional embeddings. The sequence then passed through a 12-layer Transformer encoder (each layer comprising 12 self-attention heads and GELU activations), producing an 86.6 M parameter model that achieves 83.97% top-1 accuracy on ImageNet-1k.^36^

#### Model fine-tuning

The fine-tuning of the ViT model was conducted in two stages to efficiently explore spectrogram parameter configurations while managing computational cost on an imbalanced cough/non-cough dataset.

##### Stage 1: Pilot fine-tuning on a 10% subset

A preliminary experiment was run on a randomly selected 10% subset of the full training, validation and test splits. Crucially, for each spectrogram parameter set, samples were drawn from the same underlying cough and non-cough events to ensure that performance differences reflected the spectrogram settings rather than data variation. Each configuration was fine-tuned and evaluated using identical training schedules and early stopping criteria. The F1 score was used to assess the configurations, and the one with best overall performance was selected for the next stage.

##### Stage 2: Full-scale fine-tuning on the complete dataset

The leading spectrogram configuration identified in stage 1 was then used to fine-tune the ViT model on the entire training and validation sets. Training proceeded with slightly adjustments (weight-decay) from in the pilot was applied to all available data to maximise representation of rarer cough events.

##### Subject- and diagnostic-level performance analysis

To obtain an unbiased estimate of generalisation, the fully fine-tuned model was evaluated on the test set. Beyond aggregate metrics, performance was analysed at the granularity of individual subjects and diagnostic categories. For each subject and each diagnostic label, a confusion matrix was constructed, from which sensitivity, specificity, precision and F1 score were computed. This detailed breakdown highlights how well the model discriminates cough events across different patient profiles and pathologies, revealing strengths and potential failure modes that aggregate statistics alone might obscure.

#### Definitions of evaluation metrics

The evaluation metrics mentioned before are defined as follows:

- Sensitivity (Recall): Measures the model's ability to correctly identify true positive cases (cough events).
$$\mathrm{Sensitivity}=\frac{\mathrm{True}\;\mathrm{Positive}\;(\mathrm{TP})}{\mathrm{True}\;\mathrm{Positive}\;(\mathrm{TP})+\mathrm{False}\;\mathrm{Negative}\;(\mathrm{FN})}$$
- True Positives (TP): The number of cough events correctly identified by the model.
- False Negatives (FN): The number of cough events the model incorrectly classified as non-cough events.

Sensitivity reflects the proportion of actual cough events that were correctly detected.

- Specificity: Assesses the model's ability to correctly identify true negative cases (non-cough events).
$$\mathrm{Specificity}=\frac{\mathrm{True}\;\mathrm{Negative}\;(\mathrm{TN})}{\mathrm{True}\;\mathrm{Negative}\;(\mathrm{TN})+\mathrm{False}\;\mathrm{Positive}\;(\mathrm{FP})}$$
- True Negatives (TN): The number of non-cough events correctly identified by the model.
- False Positives (FP): The number of non-cough events the model incorrectly classified as cough events.

Specificity indicates the proportion of actual non-cough events that were correctly classified.

- Precision: Represents the accuracy of the positive predictions made by the model.
$$\mathrm{Precision}=\frac{\mathrm{True}\;\mathrm{Positive}\;(\mathrm{TP})}{\mathrm{True}\;\mathrm{Positive}\;(\mathrm{TP})+\mathrm{False}\;\mathrm{Positive}\;(\mathrm{FP})}$$

Precision reflects the proportion of predicted cough events that are indeed actual coughs.

- F1 Score: The harmonic mean of precision and sensitivity, providing a balance between the two metrics.
$$F1\;\mathrm{Score}=2\times \frac{\mathrm{Precision}\times \mathrm{Sensitivity}}{\mathrm{Precision}+\mathrm{Sensitivity}}$$

The F1 score is particularly useful in scenarios with class imbalance, as it considers both false positives and false negatives, offering a single measure of performance.

#### Hyperparameters settings

The ViT was fine-tuned under the following configuration, chosen to balance convergence speed, model stability and generalisation on an imbalanced cough/non-cough dataset:

- Batch size: 128 (per device)
  Chosen to maximise GPU throughput on an NVIDIA RTX 4090 (24 GB VRAM) at 224 × 224 resolution, yielding lower-variance gradient estimates for smoother updates while avoiding out-of-memory errors.
- Optimizer: AdamW, β_1_ = 0.9, β_2_ = 0.999, ε=1 × 10^−8^
  The AdamW variant decouples weight decay from moment estimates, leading to more stable convergence and improved generalisation when fine-tuning Transformer models.
- Weight decay: 0.001
  moderate L2 penalty discourages excessively large weights, reducing overfitting without unduly constraining the model's capacity to learn fine spectral-temporal features.
  For the pilot study, the weight decay was set to 0.01 for lower risk of overfitting.
- Learning-rate schedule
  Target: 5 × 10^−5^
  Warm-up: linear ramp from 0 to 5 × 10^−5^ over the first 10% of total training steps
  Decay: cosine schedule from 5 × 10^−5^ to 0 over remaining steps
  This combination ensures rapid initial learning on the dataset, prevents unstable updates at random initialisation, and promotes fine adjustments as training nears convergence.
- Training duration:
  Max epochs: 20
  Early stopping: patience = 5 epochs (restore best validation-loss checkpoint)
  Twenty epochs allow ample opportunity to learn complex cough patterns; early stopping halts training once validation performance plateaus, guarding against overfitting and saving compute
- Class weighting: $W_{i}=\frac{N}{2\times N_{i}}$ in the cross-entropy loss
  where $W_{i}$ is the weight assigned to class *i* (cough or non-cough), *N* the total number of samples in the dataset and $N_{i}$ the number of samples belonging to class *i*.
  By up-weighting the minority (cough) class, this scheme mitigates dataset imbalance, ensuring the model attends appropriately to rare cough events and improving detection sensitivity.

## Results

### Pilot fine-tuning

**Figure 3.**
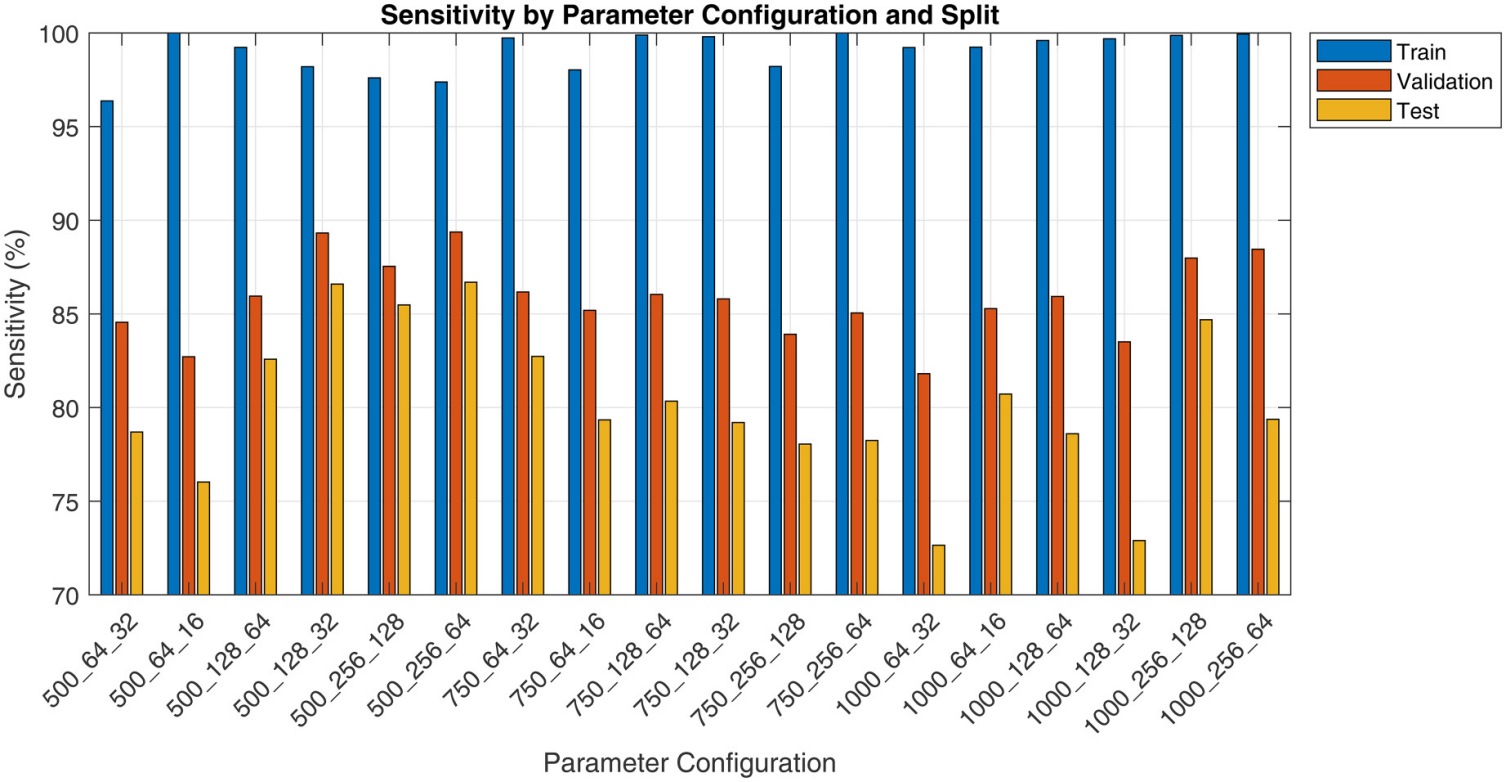
Pilot fine-tuning sensitivity by spectrogram parameter and split.

**Figure 4.**
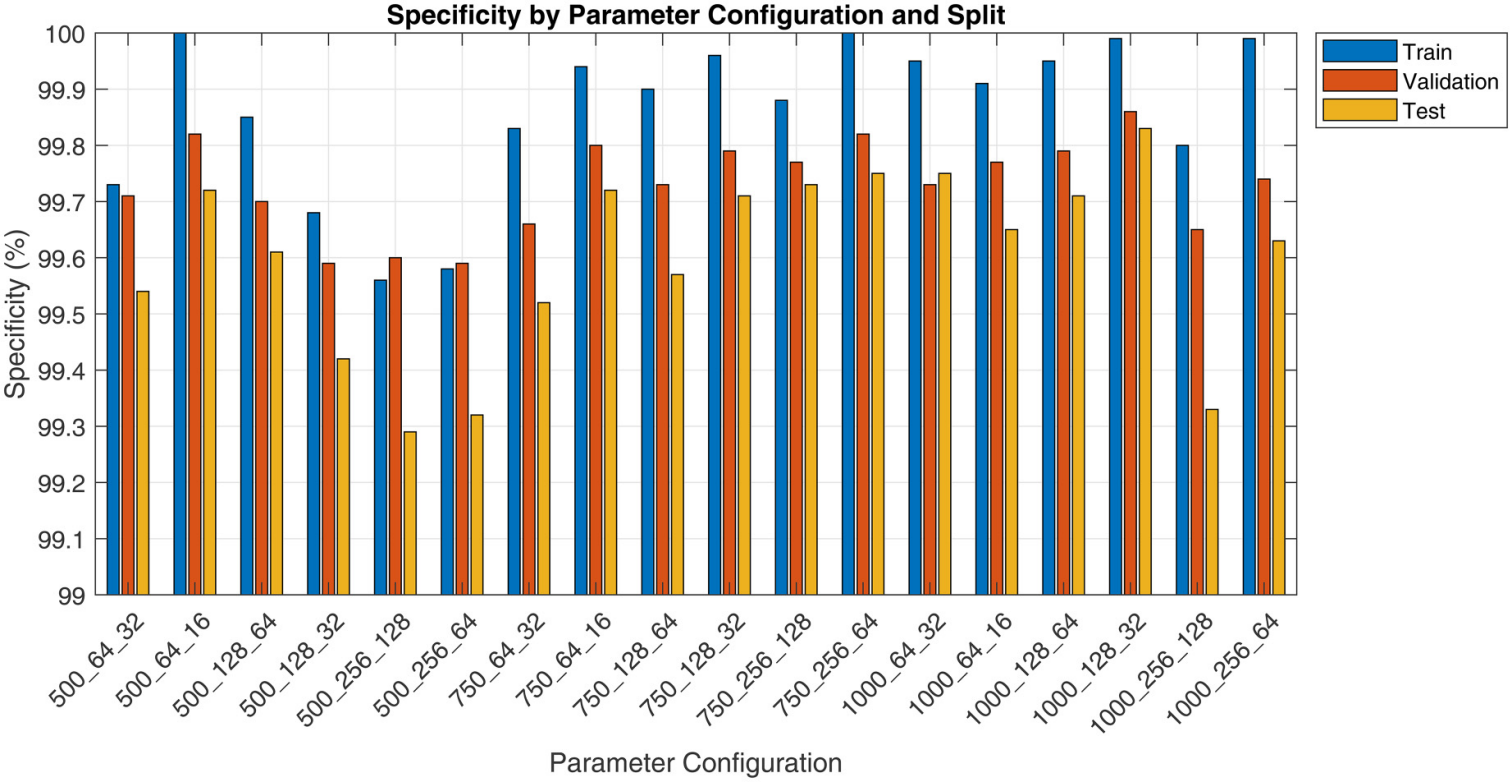
Pilot fine-tuning specificity by spectrogram parameter and split.

**Figure 5.**
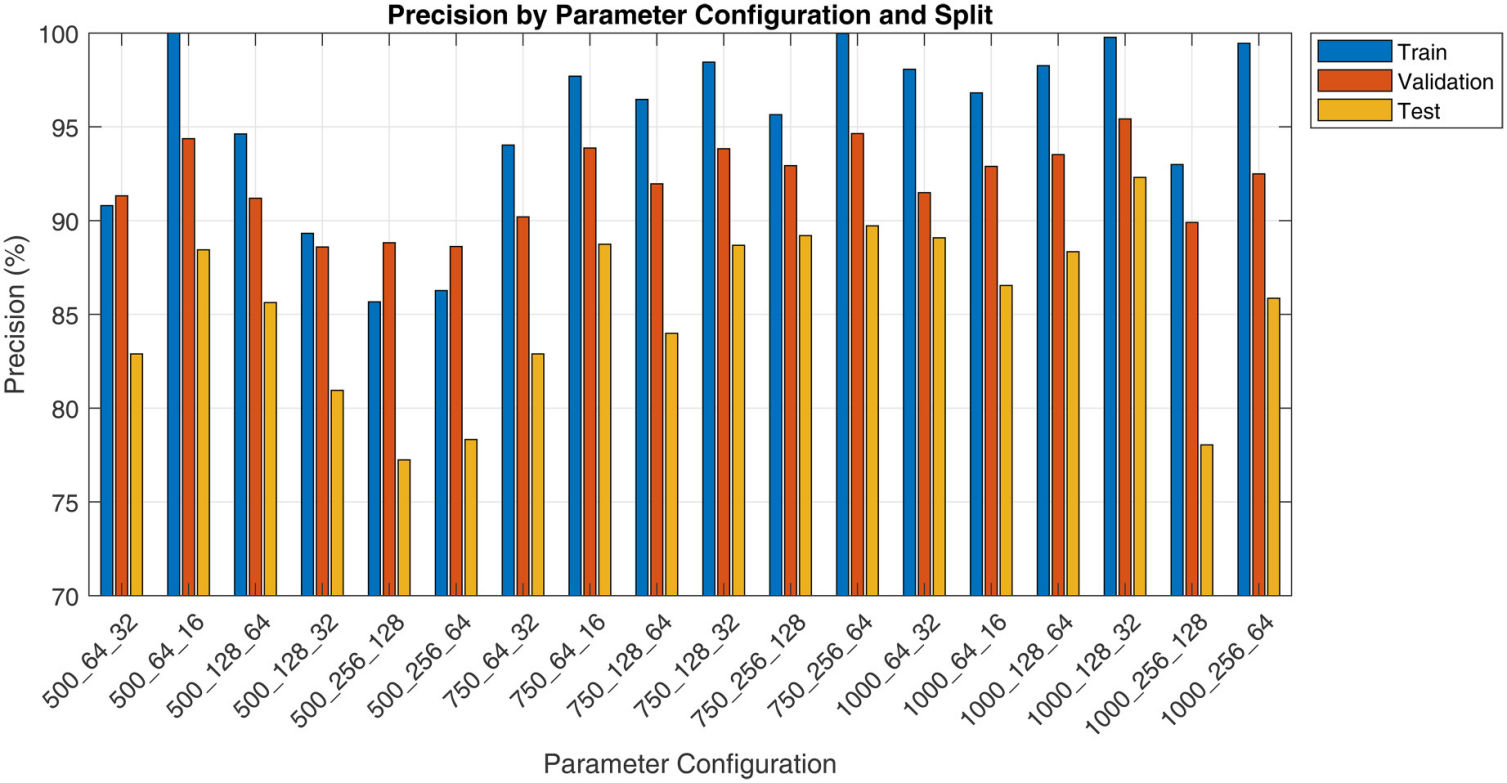
Pilot fine-tuning precision by spectrogram parameter and split.

**Figure 6.**
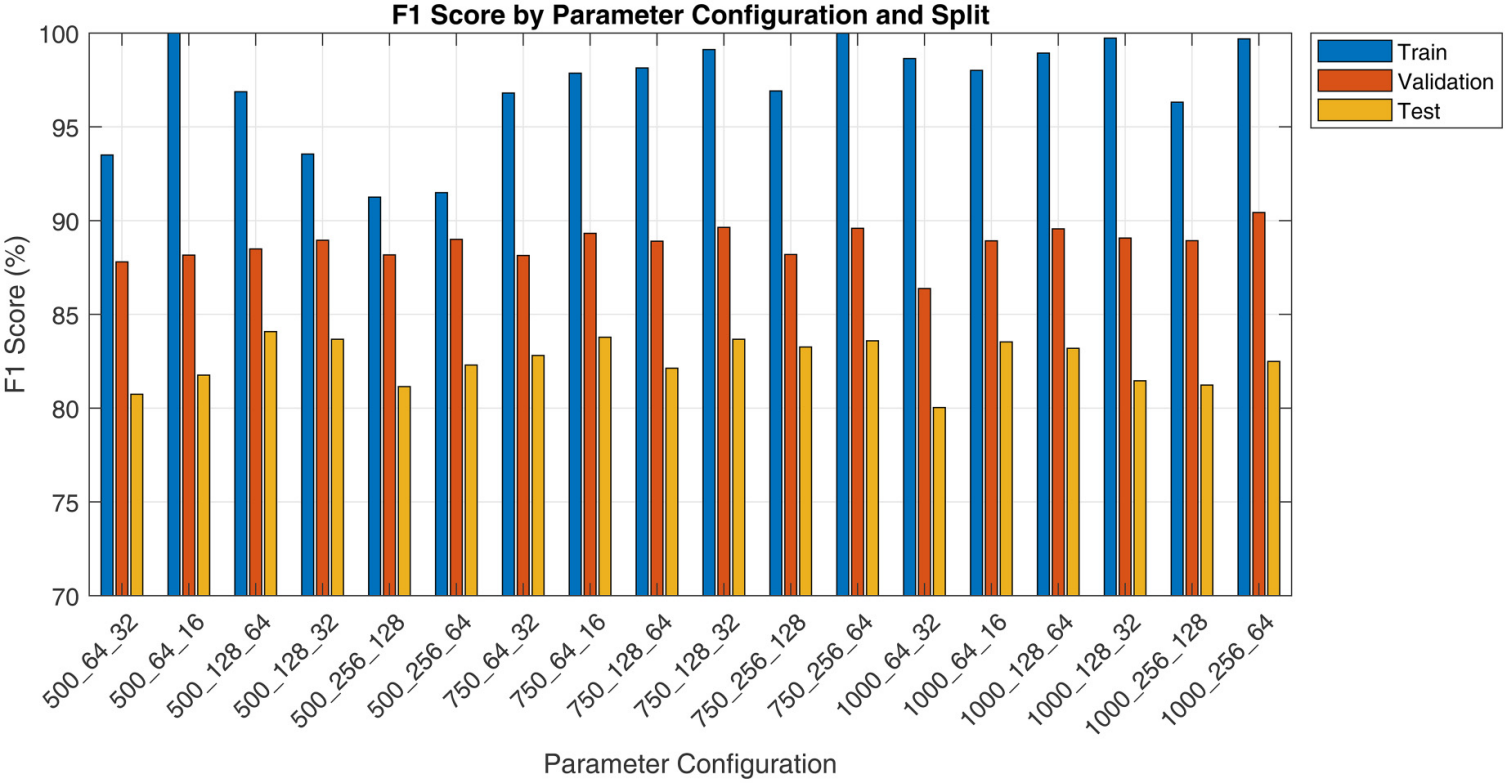
Pilot fine-tuning F1 score by spectrogram parameter and split.

The pilot study evaluated various spectrogram configurations, reported as ‘Duration_FrameLength_HopSize’, across training, validation and test sets using metrics such as sensitivity, specificity, precision and F1 score. Results are presented in Figures 3 to 6. Key findings on how segment duration, STFT frame length, and hop size influence model performance are detailed below.

Table 5 summarises the effect of varying audio segment durations (500, 750 and 1000 ms) on model performance, averaged across six STFT configurations per duration. In terms of test F1 score, the 750 ms segment duration achieved the highest average (83.21%), followed closely by the 500 ms (82.28%) and then 1000 ms (81.99%). The training F1 scores were consistently high across all durations, with 1000 ms segments achieving the highest average training F1 score (98.55%). However, the 500 ms group showed the smallest train-test gap (12.16%), suggesting less overfitting compared to longer durations.

**Table 5.** Performance metrics at different audio segment durations.

Duration	Train F1	Validation F1	Test F1	Train-test gap	Test precision	Test recall
500 ms	94.44%	88.43%	82.28%	−12.16pp	82.25%	82.68%
750 ms	98.14%	88.96%	83.21%	−14.93pp	87.20%	79.65%
1000 ms	98.55%	88.88%	81.99%	−16.56pp	86.69%	78.16%

The test recall, which indicates the model's ability to detect positive cough cases, peaked at 500 ms (82.68%) and decreased with longer durations, dropping to 78.16% at 1000 ms. Conversely, test precision increased with segment duration, reaching 86.69% and 87.20% for 1000 and 750 ms, respectively, compared to 82.25% at 500 ms.

Table 6 presents the average performance metrics for three frame length configurations, 64, 128 and 256, aggregated across all segment durations and hop sizes. Frame length 128 yielded the highest average test F1 score (83.03%), followed by 256 (82.34%) and 64 (82.11%). The model trained on frame length 256 achieved the highest test recall (82.09%) but the lowest test precision (83.07%), whereas frame length 128 balanced both recall and precision at 80.04% and 86.65%, respectively. Models trained on 64-point frames showed the greatest train-test F1 gap (15.36 pp), suggesting a higher degree of overfitting or weaker generalisation.

**Table 6.** Performance metrics at different frame lengths.

Frame length	Train F1	Validation F1	Test F1	Train-test gap	Test precision	Test recall
64	97.47%	88.12%	82.11%	−15.36 pp	86.43%	78.36%
128	97.72%	89.10%	83.03%	−14.69 pp	86.65%	80.04%
256	95.94%	89.05%	82.34%	−13.60 pp	83.07%	82.09%

Table 7 compares model performance using two common STFT hop size settings: 50% overlap (hop size = 0.5 × frame length) and 75% overlap (hop size = 0.25 × frame length). The 25% hop size yielded a higher overall F1 and test precision (86.62%). On the other hand, the 50% hop size marginally outperformed in test recall (80.42% vs 79.90%). Both hop settings produced comparable train-test gaps (14–15 pp), indicating similar generalisation behaviour.

**Table 7.** Performance metrics at different hop size.

Hop size	Train F1	Validation F1	Test F1	Train-test gap	Test precision	Test recall
50%	96.37%	88.28%	82.07%	−14.30 pp	84.14%	80.42%
25%	97.72%	89.23%	82.92%	−14.80 pp	86.62%	79.90%

Best configuration:

The configuration ‘750_128_32’ was identified as the optimal setting, achieving a strong balance between recall, precision and efficiency. It reduced the number of spectrograms generated per recording, lowering computational cost, while maintaining high detection performance. Despite an approximately 15 pp train-test gap, this was acceptable for a pilot run with only 10% of the dataset.

### Full-scale fine-tuning

The full-scale training and evaluation were carried out using the 750_128_32 spectrogram setting, identified during the pilot study as the optimal configuration. Performance was measured across training, validation and test splits using confusion matrices and standard classification metrics, as summarised in Table 8.

**Table 8.** Performance metrics of full-scale fine-tuning.

Split	TN	FP	FN	TP	Sensitivity	Specificity	Precision	F1
Train	2274484	1058	292	55645	99.48%	99.95%	98.13%	98.80%
Validation	856516	2472	2337	15968	87.23%	99.71%	86.59%	86.91%
Test	853900	2788	3478	17775	83.64%	99.67%	86.44%	85.02%

On the training set, the model achieved exceptionally high performance: sensitivity was 98.13%, specificity 99.99%, precision 99.48% and F1 score 98.80%. These results indicate the model's strong ability to both correctly detect cough events and avoid false positives during training.

In the validation set, sensitivity dropped to 86.59% and precision to 87.23%, resulting in an F1 score of 86.91%. Specificity remained high at 99.73%, suggesting reliable rejection of non-cough events.

On the test set, the model demonstrated a sensitivity of 86.44% and precision of 83.64%, yielding an F1 score of 85.02%. Specificity remained robust at 99.59%. These test results reflect a strong balance between recall and precision, with reliable generalisation performance on unseen data.

### Performance on different diagnostic categories

**Figure 7.**
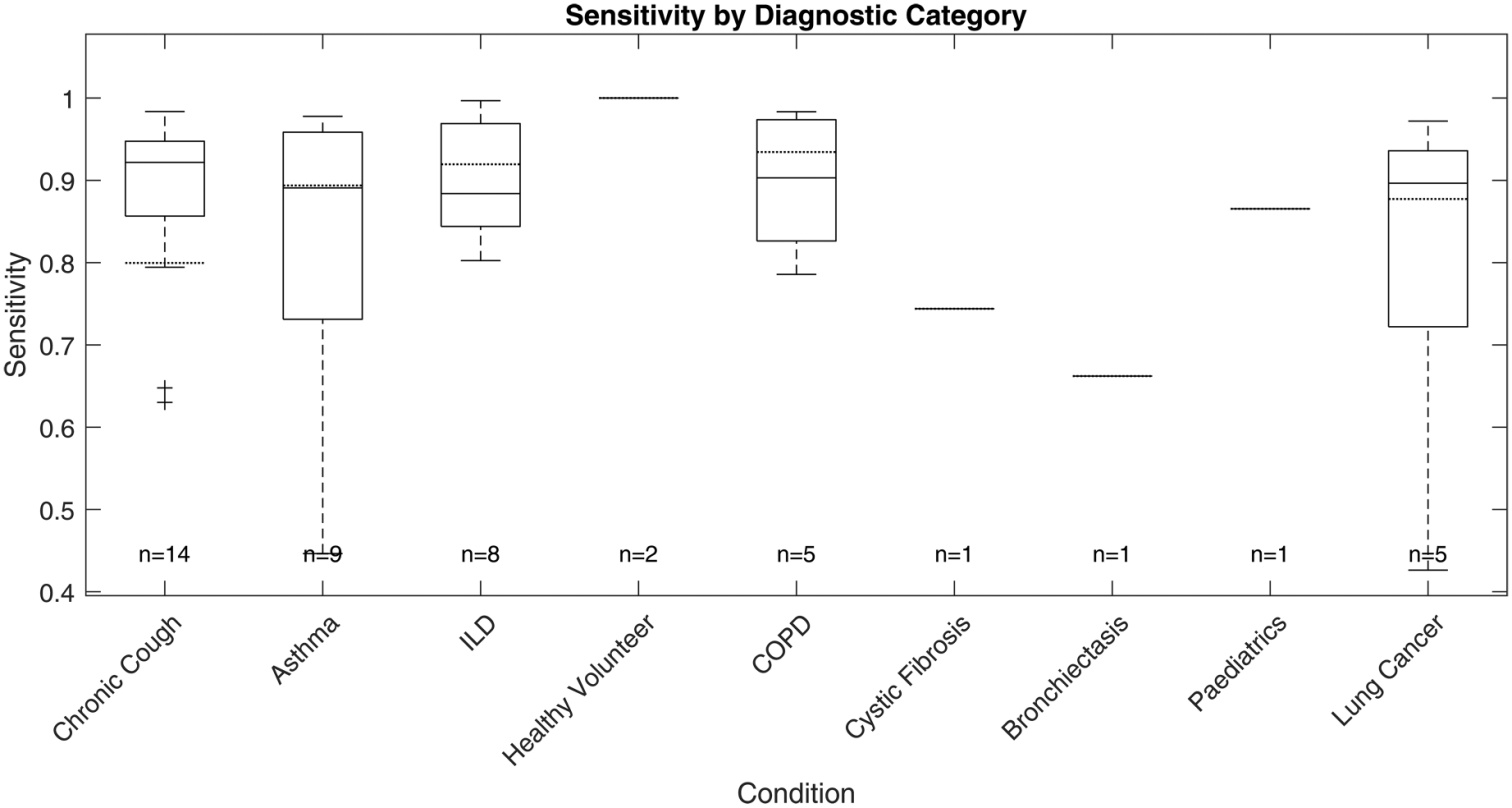
Test sensitivity boxplot by diagnostic category.

**Figure 8.**
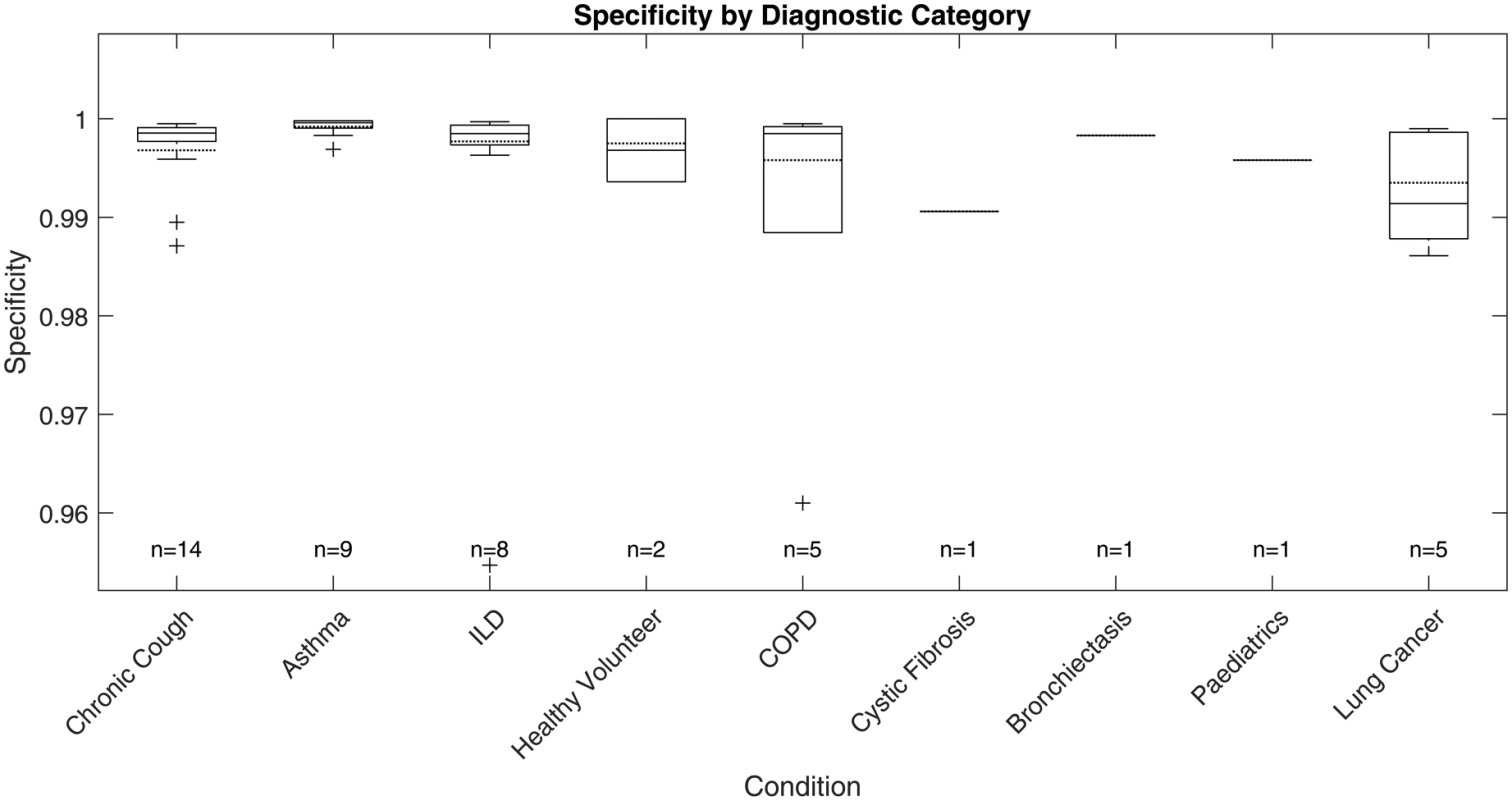
Test specificity boxplot by diagnostic category.

**Figure 9.**
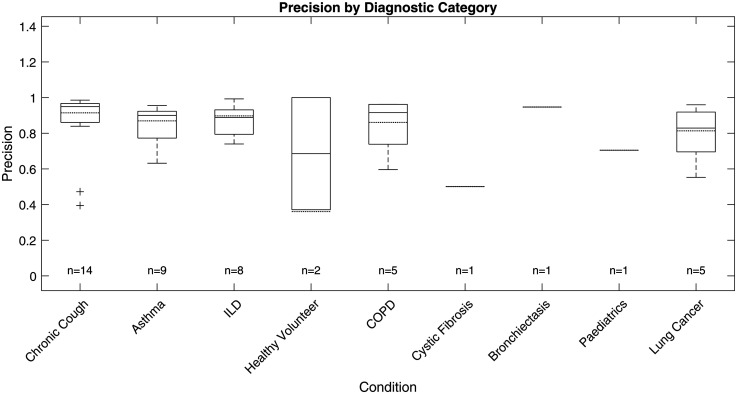
Test precision boxplot by diagnostic category.

**Figure 10.**
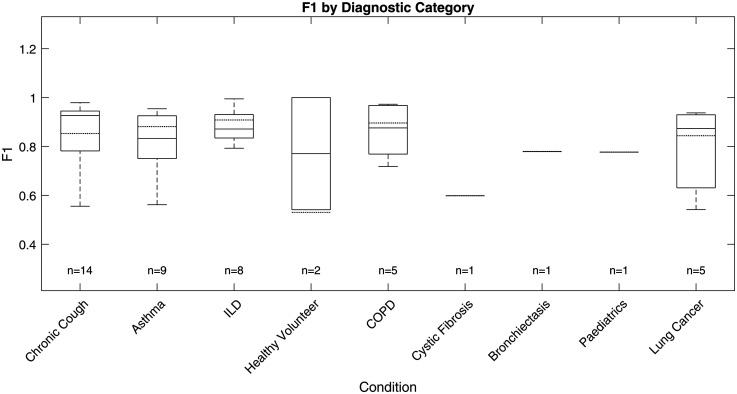
Test F1 score boxplot by diagnostic category.

**Figure 11.**
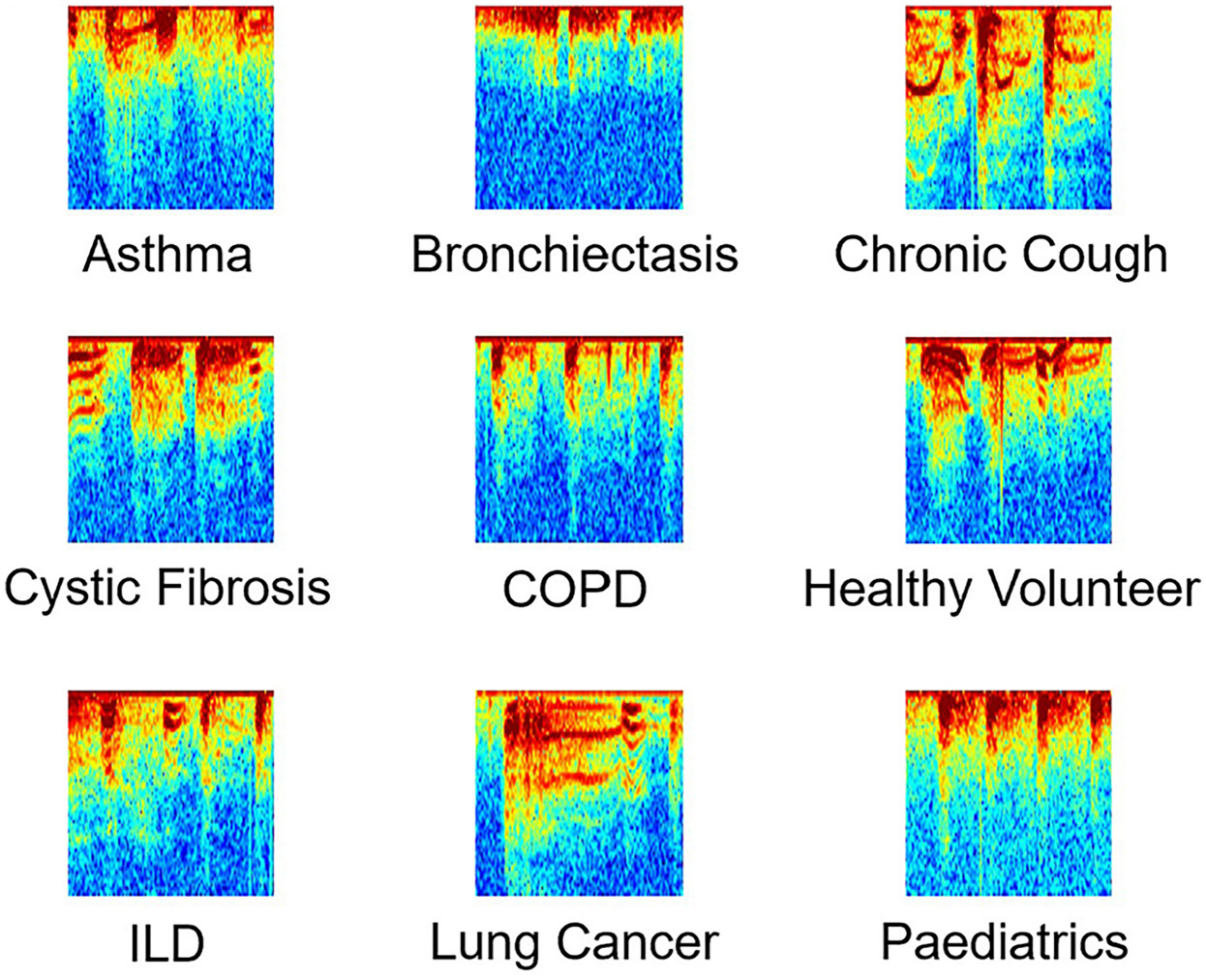
Representative processed spectrograms by disease category.

The ViT-based cough detection model demonstrated robust performance across a diverse nine-category clinical test dataset. Boxplots in Figures 7 to 10 summarise the distributions of sensitivity, specificity, precision and F1 score by diagnostic group, highlighting both inter- and intra-class variability. One healthy volunteer was excluded due to the absence of cough events. Figure 11 shows representative spectrograms for each disease category.

F1 score, the primary metric, was highest and most consistent in the ILD group (n = 8; overall F1: 88.26%, median: 87.15%, interquartile range (IQR): 83.47%–93.03%, SD: 6.81%), indicating stable detection with minimal between-subject variation. COPD (n = 5) also exhibited strong performance (overall F1: 86.37%, median: 87.59%, IQR: 76.88%–96.77%, SD: 11.16%), with limited outlier influence. Chronic cough (n = 14) showed good overall accuracy (overall F1: 85.65%, median: 92.64%), but higher variability (IQR: 78.17%–94.48%, SD: 14.01%) and some low-performing outliers (F1 < 0.6), reflecting heterogeneity in cough characteristics.

Asthma (n = 9) and lung cancer (n = 5) groups displayed broader IQRs and lower minima (asthma F1 range: 56.18%–95.43%, IQR: 75.06%–92.55%, SD: 12.94%; lung cancer F1 range: 54.18%–93.73%, IQR: 63.08%–92.96%, SD: 17.74%), suggesting greater intra-class variability and more challenging acoustic profiles. For healthy volunteers (n = 2), F1 scores varied widely (54.12%, 100%), likely due to sporadic cough occurrence. Single-subject categories (cystic fibrosis, bronchiectasis, paediatrics) yielded F1 scores of 59.84% to 77.93%, but sample sizes were too small for meaningful interpretation.

Precision and sensitivity mirrored the F1 patterns. ILD showed high precision (87.00%, SD: 8.65%) and sensitivity (89.91%, SD: 7.11%), while asthma, chronic cough and lung cancer groups exhibited greater disparities between precision and recall, with some chronic cough cases showing low precision (down to 39.47%), indicating occasional misclassification of non-cough events, and some asthma cases displaying reduced recall. Specificity was uniformly high (>99%) across all categories due to the class imbalance and is less discriminatory for inter-group comparison.

## Discussion

### Pilot fine-tuning

Our evaluation shows that spectrogram parameter choices significantly influence ViT-based cough detection.

Segment duration had a notable impact on generalisation and recall. Shorter segments (500 ms) yielded the highest recall and lowest train-test F1 gap, likely due to more training samples and focused temporal windows. However, their precision was lower, suggesting limited context. Longer segments (1000 ms) improved precision but suffered from reduced recall and overfitting, potentially due to background noise inclusion and smaller dataset size. The 750 ms duration balanced both context and sample count, producing the best average test F1.

Frame length affected the preservation of spectral features post-resizing. While all spectrograms were resized to 224 × 224, short frames (64-point) originally generated wider images that likely compressed temporal details. Long frames (256-point) led to narrow spectrograms with reduced temporal variation. The 128-point frame provided a balanced original aspect ratio, maintaining key time-frequency patterns after resizing and delivering the strongest overall performance.

Hop size determined the temporal resolution and smoothness of spectrograms. The 25% hop-generated denser time steps, improving precision and test F1, likely due to better preservation of transient acoustic cues. The 50% hop performed comparably but showed slightly lower precision, possibly due to coarser time sampling.

Among all configurations, 750 ms segment duration, 128-point frame length and 32-point hop size (25% overlap) provided the best balance between recall, precision and generalisation. This setup likely aligns well with the typical duration of cough events, preserves critical acoustic features after resizing, and offers sufficient temporal resolution. It consistently achieved strong test performance across metrics, making it the optimal choice for final model training.

### Full-scale fine-tuning

#### Sensitivity, specificity and data imbalance

The ViT model, optimised using class weighting and F1-based validation, achieved high specificity (99.67%) and good sensitivity (83.64%) on the test set. This balance indicates the model is effective at rejecting non-cough sounds and is careful to rare cough events, a common challenge in imbalanced datasets. Class weighting in the loss function was particularly valuable for discouraging the model from biasing towards the dominant (non-cough) class.

#### Recall, precision and F1 score

By using F1 score for validation loss and early stopping, training directly optimised the trade-off between recall (83.64%) and precision (86.44%), leading to a strong overall test F1 (85.02%). This approach ensures the model remains effective in both identifying coughs and limiting false positives, which is essential for real-world usability. However, the recall metric suggests that some true coughs are still missed, highlighting an area for further improvement.

#### Train-test and validation-test gaps

The observed gap between training and test F1 scores (98.80% vs 85.02%) suggests that, despite regularisation strategies such as weight decay and early stopping, some overfitting persists. While the model demonstrates a strong ability to fit the training data, its generalisation to unseen examples is more limited. This is a common challenge in deep learning, especially with imbalanced and acoustically diverse audio datasets.

### Performance analysis on different diagnostic categories

The ViT-based model exhibited differential performance across diagnostic categories, highlighting both its clinical potential and the challenges of automated respiratory sound analysis. Stable, high F1 scores in ILD and COPD suggest these conditions produce acoustically consistent coughs, likely reflecting characteristic pathophysiology. In contrast, increased intra-group variability and outlier performance in asthma and lung cancer underscore the impact of heterogeneous disease presentation, variable symptom severity and potential confounding from background noise or comorbidities, complicating robust detection.

Variability within larger or clinically diverse groups, such as chronic cough and asthma, further emphasises the need for models resilient to both disease-specific and inter-individual differences. This highlights the importance of diverse, representative datasets and may point towards the future utility of stratified or personalised modelling approaches in heterogeneous populations.

Although greater sample sizes tended to enhance performance stability, persistent variability in certain categories indicates that mere increases in subject numbers are insufficient without addressing underlying acoustic and clinical diversity. Strategic, clinically informed data sampling and targeted collection are thus crucial for improving model generalisability.

Given the substantial class imbalance, specificity remained uniformly high but is less informative for clinical evaluation. Precision and recall are more relevant, as they directly measure the model's ability to accurately identify true cough events without excessive false positives; notable metric disparities between categories further support the need for nuanced, context-specific assessment.

Overall, these findings demonstrate that ViT-based spectrogram analysis offers reliable cough detection in certain clinical groups, but further work—potentially including adaptive thresholds or multimodal approaches—will be required to ensure robust performance in diagnostically heterogeneous or low-event-rate populations.

### Comparisons to other studies

To our knowledge, this is the first study to systematically investigate how STFT parameter settings affect machine learning-based cough detection performance using image classification models, specifically with a ViT architecture. We developed a fully automated system validated on a large and diagnostically diverse dataset, collected using a standardised protocol with contact microphones (VitaloJAK^15,18^), which ensures signal consistency and reliability. Our dataset comprises 47 participants spanning nine diagnostic categories, making it the most extensive and varied cohort among comparable studies. Importantly, all data were collected in controlled clinical settings rather than through crowdsourcing, further supporting the robustness of our results. Table 9 shows the performance comparisons to other notable studies.

**Table 9.** Comparisons to other cough detection studies.

Study	Fully automated	Data collection	Audio format	Feature extraction	Classifica-tion model	Subjects	Sensitivity	Specificity	Precision
LCM^12^	No, manual calibration required	Necklace microphone	16 kHz 64 kbps	MFCC	HMMs	n = 15	91%	99%	-
SIVA^19^	Yes	Necklace microphone	Unknown	Unknown	DNN	n = 27	Day 88.6% / Night 84.2%	99.97%	Day 89.7% / Night 87.1%
Hyfe^20^	Yes	Smartwatch microphone	Unknown	Unknown	CNN	n = 23	90.4%	-	87.5%
This study	Yes	VitaloJAK Contact microphone	8 kHz 16 bit	Spectrogram	ViT	n = 47	83.64%	99.67%	86.44%

CNNs: convolutional neural networks; LCM: Leicester Cough Monitor; MFCCs: mel-frequency cepstral coefficients; HMM: hidden Markov model; ViT: Vision Transformer.

When compared to LCM,^12^ our system shows lower sensitivity but superior specificity. This trade-off is expected given the considerably more imbalanced and diagnostically heterogeneous dataset we used. Notably, the LCM system required semi-manual threshold adjustments for each participant, whereas our workflow is entirely automated and uniformly applied, enhancing its scalability and real-world utility. LCM's participant group was also limited to 15 individuals, in contrast to our broader and more inclusive cohort.

SIVA^19^ reported marginally higher performance metrics; however, their paper omits crucial details such as the number of positive and negative samples, preventing any assessment of class imbalance, an essential factor in evaluating classifier performance. Their model was tested on only 27 subjects across 4 diagnostic categories and provides minimal transparency regarding the deep neural network architecture, preprocessing techniques or training protocols.

Similarly, Hyfe's system^20^ achieved slightly higher sensitivity and precision using a CNN on unspecified ‘spectral’ inputs. However, their technical documentation lacks sufficient detail on feature extraction, model structure or inference mechanics. Moreover, with just 23 participants, though from nine diagnostic groups, Hyfe's study remains limited in size, and again, the class distribution is not disclosed, raising concerns about performance overestimation due to potential data imbalance.

In summary, our study contributes a comprehensive and transparent framework for cough detection using spectro-temporal analysis and transformer-based classification. Compared with prior works, we used a larger and more diagnostically diverse participant pool, adopted a rigorously standardised data collection setup, and provided full technical disclosure of our preprocessing and modelling workflow. Despite working with more challenging, imbalanced data, our model achieved competitive results in sensitivity, specificity, and precision. We also emphasise the importance of transparency in reporting dataset characteristics, especially class imbalance and technical details, as these are often underreported in the field. Addressing these issues head-on, rather than omitting them, is crucial for fostering reproducibility, benchmarking progress and driving meaningful innovation in automated respiratory health monitoring.

### Cross-validation analysis

To further evaluate the robustness and generalisability of the model, a five-fold cross-validation was performed on the full-scale fine-tuning dataset. In the original fine-tuning, the data splits were carefully curated to maintain similar diagnostic category and demographic distributions across subsets, and all spectrograms from the same subject were grouped within a single subset to prevent data leakage (section Data split).

However, given the uneven category distribution and limited sample size of certain diagnostic groups, it was not practical to split the dataset into five folds while preserving both balanced category representation and subject-level separation. As a result, a sample-based randomisation method was adopted to generate the folds. This approach inevitably led to some highly similar cough samples from the same subject appearing across both training and validation sets, introducing a mild degree of data leakage.

The 5-fold cross-validation achieved an average F1 score of 93.03% ± 0.17%. Although the cross-validation results showed moderately higher performance metrics compared with the original fine-tuning, this improvement was primarily attributed to the data overlap rather than genuine enhancement in model generalisation. Therefore, while the cross-validation outcomes provide additional support for the model's stability, the subject-level split results were retained as the final performance metrics, as they better reflect real-world generalisation and avoid potential biases introduced by intra-subject similarity.

### Observations

This study was necessarily constrained by the availability of computational resources and dataset sizes, which limited the scope of experiments that could be conducted. The work focused specifically on binary cough versus non-cough discrimination, rather than exploring more fine-grained classification tasks such as distinguishing between diagnostic categories or labelling cough events with greater temporal precision. While steps were taken to reduce issues of class imbalance and overfitting, rare diagnostic categories and edge cases remained particularly challenging for the automated models.

Furthermore, the study explored only a limited range of preprocessing and modelling choices. Additional spectrogram parameters, such as varying the STFT size, trying alternative window functions (e.g. Hamming or Blackman), or experimenting with different resizing and transformation methods, could provide further performance gains. Similarly, more diverse data augmentation strategies and the use of soft labelling for ambiguous cough segments could improve generalisation and labelling accuracy. Beyond this, exploring alternative acoustic representations such as mel spectrograms, MFCCs, or wavelet-based scalograms may offer complementary insights. Finally, the models developed here were not optimised for computational efficiency; designing more compact architectures would be essential for enabling real-time implementation on mobile or wearable devices.

## Conclusion

This study presents a comprehensive process for automated cough detection using spectro-temporal analysis and ViT architecture. By systematically exploring STFT parameter configurations and utilising a well-curated, clinically diverse dataset, we demonstrate that ViT-based models can achieve high precision, recall and generalisation across a wide range of respiratory conditions. The optimal configuration (750 ms segment duration, 128-point frame length and 32-point hop size) balances temporal context and spectral detail, yielding a robust classifier capable of handling class imbalance and acoustic variability.

Our results demonstrate the potential of transformer-based models in medical audio classification tasks, particularly when paired with carefully designed preprocessing and standardised data collection. Unlike prior systems that relied on manual calibration, opaque processing steps, or limited datasets, our workflow offers a transparent and scalable solution suitable for further development and deployment.

Future work should focus on extending this model to enable disease-specific cough detection, which may enhance diagnostic precision by recognising condition-specific acoustic patterns. Additionally, exploring the influence of patient demographics, including age and sex, on model performance could reveal biases or generalisation limitations, guiding more appropriate model design. Finally, the feasibility of real-time implementation should be investigated, particularly for integration with portable devices such as smartphones or wearables, enabling continuous and unobtrusive cough monitoring in everyday environments.

By addressing these directions, we aim to advance the clinical utility of automated cough detection systems and contribute meaningfully to the broader field of digital respiratory health monitoring.

## Supplemental Material

sj-xlsx-1-dhj-10.1177_20552076251394623 - Supplemental material for Cough sound spectro-temporal analysis and automated detection using Vision Transformers Supplemental material, sj-xlsx-1-dhj-10.1177_20552076251394623 for Cough sound spectro-temporal analysis and automated detection using Vision Transformers by Keming Tan, Jacky Smith and Patrick Gaydecki in DIGITAL HEALTH
